# Ectopic ACTH-producing neuroendocrine tumor occurring with large recurrent metastatic pheochromocytoma: a case report

**DOI:** 10.1186/s12902-022-01090-8

**Published:** 2022-07-19

**Authors:** Fumi Saishouji, Sarie Maeda, Hideaki Hamada, Noriko Kimura, Ai Tamanoi, Saiko Nishida, Masaji Sakaguchi, Motoyuki Igata, Kiho Yokoo, Fumi Kawakami, Eiichi Araki, Tatsuya Kondo

**Affiliations:** 1 Department of Diabetes, Metabolism and Endocrinology, Kumamoto University Hospital, 1-1-1 Honjo, Chuo-Ward, Kumamoto, 860-8556 Japan; 2 Department of Diagnostic Pathology, Department of Clinical Research, National Hospital Organization, Hakodate Hospital, 16-18 Kawahara, Hakodate, Hokkaido 041-8512 Japan; 3 Department of Metabolic Medicine, Faculty of Life Sciences, Kumamoto University, 1-1-1 Honjo, Chuo-Ward, Kumamoto, 860-8556 Japan; 4 Department of Diagnostic Pathology, Kumamoto University Hospital, 1-1-1 Honjo, Chuo-Ward, Kumamoto, 860-8556 Japan

**Keywords:** Ectopic ACTH-producing tumor, Recurrent metastatic pheochromocytoma,, Cortisol, Catecholamine,, Positive feedback loop

## Abstract

**Background:**

Ectopic ACTH-dependent Cushing syndrome is rarely caused by pheochromocytoma (PCC). Glucocorticoid-regulated positive feedback loops in ACTH and catecholamines were proposed in some similar cases.

**Case presentation:**

We present here an 80-year-old man who had previously undergone surgery for a left adrenal PCC and newly developed severe hypertension, hypokalemia, and typical Cushingoid manifestations. Investigations revealed hyperglycemia, hypokalemia, and extremely high catecholamines and their metabolites, ACTH and cortisol. Imaging modalities showed a recurrent large left adrenal mass positively visualized with ^123^I-metaiodobenzylguanidine as well as somatostatin receptor scintigraphy. Surgical interventions were not indicated; thus, metyrapone, phentolamine, and doxazocin were initiated, which successfully controlled his symptoms and biochemical conditions. With the evidence that metyrapone administration decreased ACTH and catecholamine levels, the existence of positive feedback loops was speculated. During the terminal stages of the disease, additional metyrosine treatment successfully stabilized his physiological and biochemical conditions. Upon the patient’s death, pathological autopsy was performed. Immunohistochemical analysis indicated that the tumor appeared to be co-positive with tyrosine hydroxylase (TH) as well as ACTH in most tumor cells in both PCC and liver metastasis. Most cells were clearly positive for somatostatin receptor 2 staining in the membrane compartment. The dense immunostaining of ACTH, TH, dopamine-β-hydroxylase and the large tumor size with positive feedback loops may be correlated with high levels of ACTH and catecholamines in the circulation.

**Conclusions:**

We experienced a case of severe ectopic ACTH producing the largest reported recurrent malignant left PCC with liver metastases that presented positive feedback loops in the ACTH/cortisol and catecholamine/cortisol axes. Clinicians should be aware of the paradoxical response of ACTH on metyrapone treatment and possible steroid-induced catecholamine crisis.

## Background

Pheochromocytomas (PCCs) or paragangliomas are rare neuroendocrine tumors (NETs) that typically arise in chromaffin tissue, with an overall incidence of 0.4–2.1 cases per million people [1]. Cushing’s syndrome caused by ectopic ACTH-producing NET is also a considerably rare disease. Simultaneous production of catecholamine and ACTH is even rarer, ranging from 3 to 25% of cases of ectopic ACTH syndrome [2–4]. The major sources of ectopic ACTH production are bronchial carcinoid tumors (36–43%), lung cancers (18–20%), and medullary thyroid cancers (3–7%) [5]. Although the negative feedback regulation of ACTH/cortisol and the positive regulation of the catecholamine/cortisol axis are well understood, paradoxical ACTH upregulation under hypercortisolemia is observed in some ectopic ACTH-producing PCCs [6–8]. The etiology of these two endocrine manifestations, such as ectopic ACTH production and PCC, is not well understood.

In this report, we describe the case of a large ectopic ACTH-producing recurrent malignant PCC in the left adrenal gland with liver metastases, presenting possible ACTH-driven hypercortisolemia and hypercatecholaminemia.

## Materials and methods

This case study was conducted according to the CARE guidelines [9]. Written informed consent was obtained from the son of the patient.

### Pathological and immunohistochemical analysis.

The dissected tumor tissues on autopsy were fixed in 10% buffered formalin, embedded in paraffin, and sliced into 3-μm-thick tissue sections for histological analyses. These sections were subjected to hematoxylin and eosin staining and immunohistochemistry. Whole sections of the representative region were submitted for immunohistochemistry. The procedures and the information of used antibodies were described elsewhere [10]. Anti-ACTH (mouse monoclonal (02A3), 1:100, DAKO) antibody was also used. In particular, somatostatin receptors (SSTRs) were examined by fluorescent immunohistochemistry. Primary antibodies against SSTRs (anti-SSTR1 (rabbit monoclinal (ab137083), 1:100; Abcam, anti-SSTR2 (rabbit monoclinal (ab134152), 1:100; Abcam, anti-SSTR3 (rabbit monoclinal (ab137026), 1:100; Abcam, anti-SSTR3 (rabbit monoclinal (ab109495), 1:100; Abcam) and secondary antibodies (Alexa Fluor 488 or 555 (Molecular Probes, Eugene, OR, USA)) were used. The pictures in Figs. 4 and 5 were taken by microscope BIOREVO BZ-9000 system (Keyence, Osaka, Japan) at the resolution of 1360 × 624 pixels. Objective lens of Plan Fluor ELWD DM × 20 NA 0.45 creating × 200 magnification was used to take pictures on monochrome manner using BZ-II observation application and pseudo-color green (SSTR2) or blue (DAPI) was used. Any processing or enhancement were not applied to those pictures upon merge.

The human adrenal medulla was stained in parallel as a positive control for TH, DBH and CgA, and the human pituitary gland was stained as a positive control for ACTH.

### Statistical analysis

When comparing the values of ACTH, cortisol, adrenaline, noradrenaline, dopamine, urinary metanephrine and urinary normetanephrine before (off) and after (on) treatment with metyrapone, we employed paired *t*-test for the statistical analysis using SPSS 16.0 software (IBM Corp., Armonk, NY, USA), because the number of samples is small (*n* = 3 in each group). Two-sided *p*-values of < 0.05 were considered to indicate statistical significance.

## Case presentation

**Fig. 1:**
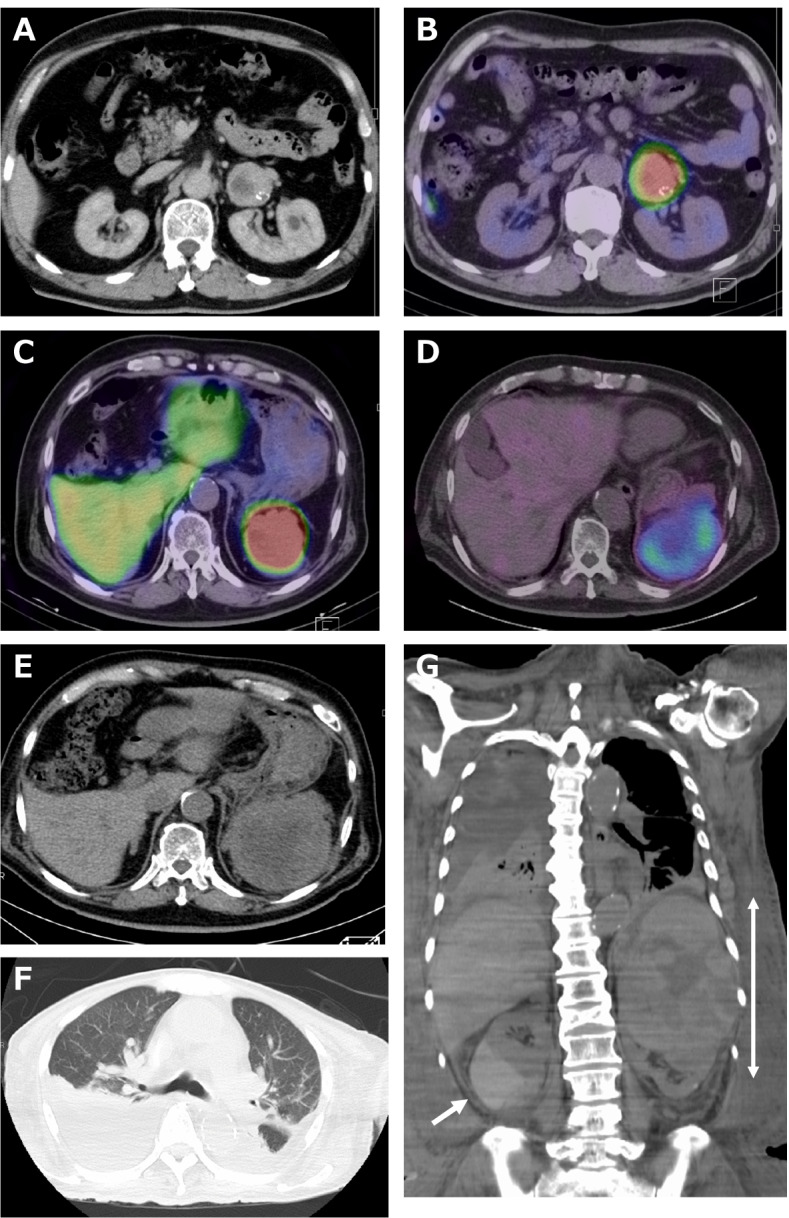
Representative images during the clinical course**. A**: CT image at the diagnosis of sole PCC. **B**: ^123^I-MIBG scintigraphy image at the diagnosis of sole PCC. **C**: ^123^I-MIBG scintigraphy image at the recurrence of PCC 7 years after surgery. **D**: SRS image at the diagnosis of recurrent PCC with simultaneous ACTH production. E–G: CT images at the terminal stage. Arrow in G indicates right adrenal subcortical bleeding. Double arrow in G indicates the maximum tumor diameter. MIBG, metaiodobenzylguanidine; SRS, somatostatin receptor scintigraphy

A 69-year-old man presenting severe hypertension (170 ~ 180/90 ~ 100 mmHg) and an incidentaloma was diagnosed with left adrenal PCC due to mild increased levels of serum catecholamines (adrenaline (AD) 30 pg/mL (< 100); noradrenaline (NAD) 690 ng/mL (100 ~ 450); dopamine (DA) 10 pg/mL (< 20)) and their urinary metabolites (U-metanephrine (U-MT) 0.22 mg/day (0.04 ~ 0.19); U-normetanephrine (U-NMT) 5.94 mg/day (0.09 ~ 0.33)), and a 42-mm tumor in the left adrenal gland (Fig. 1 A), with accumulation of ^123^I-metaiodobenzylguanidine (MIBG) scintigraphy (Fig. 1 B) 11 years ago. The patient had no family history of endocrine disorders, including multiple endocrine neoplasia 2A. At that time, circadian variations of ACTH and cortisol were maintained (ACTH 6.63 pg/mL, cortisol 3.4 μg/dL (< 5.0) at 23:00). Dexamethasone 1 mg test successfully suppressed both ACTH (4.68 pg/mL) and cortisol (2.0 μg/dL (< 5.0)). Urinary cortisol excretion was 34.4 μg/day (11 ~ 80), suggesting that obvious hypercortisolemia was not evident biochemically at that time.

Thus, left adrenalectomy was performed successfully, hypertensive symptoms and biochemical abnormalities in catecholamine levels were totally dissolved, and the patient was followed up by yearly medical checkup. 7 years later, a recurrent tumor (68 mm) was detected in the splenic hilum, which was diagnosed as a recurrent PCC due to increased levels of serum catecholamines (AD 36 pg/mL (< 100); NAD 1244 pg/mL (100 ~ 450); DA 37 pg/mL (< 20)) and their urinary metabolites (U-MT 0.25 mg/day (0.04 ~ 0.19); U-NMT 5.80 mg/day (0.09 ~ 0.33)) and accumulation of ^123^I-MIBG scintigraphy (Fig. 1 C). At that time, since the patient refused any further treatments including surgery, α-blocker (gradual increase of doxazocin up to 6 mg) was implemented.

Two years ago, the patient was urgently admitted to our hospital with the complaints of hypertension and nausea. Hyperglycemia (random glucose 294 mg/dL (73 ~ 109), HbA1c 10.0% (4.9 ~ 6.0), glycated albumin 29.2% (11.0 ~ 16.0)) and physical Cushingoid manifestations such as moon face, flashing, thin skin, edema, and abdominal striae became apparent. There was no skin hyperpigmentation. On examination, he was hypertensive (162/60 mmHg). His heart rate was 77 beats per minutes with a regular pattern under the administration of α-blocker (doxazocin 10 mg), β-blocker (bisoprolol 2.5 mg), calcium channel antagonist (nifedipine 40 mg), and angiotensin II receptor antagonist (telmisartan 40 mg). His BMI was 29.2 kg/m^2^. Adrenal CT scan revealed an enlarged recurrent left adrenal tumor (91 mm). The tumor was densely positive with ^123^I-MIBG scintigraphy (not shown) as well as mildly positive with somatostatin receptor scintigraphy (^111^In-pentetreotide. SRS; Fig. 1 D). Notably, SRS was not positive in liver metastatic lesions, which were diffusely evident on plain CT images. Biochemical examination revealed severely high levels of catecholamines (AD 13,071 pg/mL (< 100); NAD 8987 pg/mL (100 ~ 450); DA 190 mg/mL (< 20)) as well as extremely high ACTH (1402 pg/mL (7.2 ~ 63.3)) and cortisol (78.4 μg/dL (7.07 ~ 19.6)), with a high ACTH/cortisol ratio (17.9 × 10^− 4^), suggesting ACTH-driven hypercortisolemia (Table. 1). Serum potassium was 2.1 mEq/L (3.6 ~ 4.8) and the counts of circulating eosinophil was totally suppressed at 0.0% (0.4 ~ 8.6) (Table. 1). A diagnosis of ACTH-producing recurrent malignant metastatic PCC was made on the basis of rapidly developing clinical symptoms, biochemical evidence of catecholamine, ACTH, and cortisol excess, and the appearance of a left adrenal mass with liver metastases on CT and radio isotope imaging. Circadian fluctuations of ACTH and cortisol were blunted (ACTH 421.2 pg/mL, cortisol 13.0 μg/dL (< 5.0) at 23:00). High-dose dexamethasone suppression test (8 mg) was not performed because brain MRI and SRS were both negative in the pituitary gland (data not shown) and a positive feedback loop in the ACTH/cortisol axis was suspected because of the high ratio of ACTH/cortisol (17.9 × 10^− 4^).

**Table 1:** Routine laboratory data upon administration

Complete Blood Count		Blood chemistory		Tumor markers	
WBC	8400 /μL (3300 ~ 8600)	T-P	4.8 g/dL (6.6 ~ 8.1)	PSA	1.950 ng/mL (< 4.0)
RBC	4.42 × 10^6^/μL (4.4 ~ 5.6)	Alb	2.8 g/dL (4.1 ~ 5.1)	NSE	2.1 ng/mL (< 16.3)
Hb	13.9 g/dL (13.7 ~ 16.8)	T-Bil	2.1 mg/dL (0.4 ~ 1.5)		
Ht	39.2% (40.7 ~ 50.1)	AST	1 U/L (13 ~ 30)		
MCV	88.7 fL (83.6 ~ 98.2)	ALT	47 U/L (10 ~ 42)		
MCH	31.4 pg (27.5 ~ 33.2)	LD	749 U/L (124 ~ 222)		
MCHC	35.5 g/dL (31.7 ~ 35.3)	γ-GTP	89 U/L (13 ~ 64)		
Plt	81 × 10^3^/μL (158 ~ 348)	CHE	194 U/L (240 ~ 486)		
Neutro	95.4% (38.5 ~ 80.5)	CK	216 U/L (59 ~ 248)	**Urine data**	
Baso	0.1% (0.2 ~ 1.4)	LDL-C	46 mg/dL (65 ~ 163)	Glu	(3+)
Eosino	0.0% (0.4 ~ 8.6)	HDL-C	60 mg/dL (38 ~ 90)	Pro	(3+)
Lymph	3.1% (18.2 ~ 47.7)	TG	130 mg/dL (40 ~ 234)	OB	(−)
Mono	1.4% (3.3 ~ 9.0)	BUN	21.6 mg/dL (8 ~ 20)	Nit	(−)
		Cre	1.13 mg/dL (0.7 ~ 1.1)	Ket	(−)
		eGFR	49 (> 60)	Bil	(−)
**Endocrinological exam.**		Na	141 mEq/L (138 ~ 145)		
ACTH	1402 pg/mL (7.2 ~ 63.3)	K	2.1 mEq/L (3.6 ~ 4.8)		
Cortisol	8.4 μg/dL (7.07 ~ 19.6)	Cl	91 mEq/L (101 ~ 108)	U-TP	1712.0 mg/day (< 150)
TSH	0.30 μIU/mL (0.35 ~ 4.94)	Ca	7.6 mg/dL (8.8 ~ 10.1)	U-cortisol	137.3 μg/day (11 ~ 80)
F-T3	< 1.50 pg/mL (1.88 ~ 3.18)	IP	3.3 mg/dL (2.7 ~ 4.6)	U-MT	76.0 mg/day (0.04 ~ 0.19)
F-T4	0.58 ng/dL (0.70 ~ 1.48)	UA	7.1 mg/dL (3.7 ~ 7.8)	U-NMT	79.8 mg/day (0.09 ~ 0.33)
AD	13,071 pg/mL (< 100)	Glucose	294 mg/dL (73 ~ 109)		
NAD	8987 pg/mL (100 ~ 450)	CPR	2.4 ng/mL (0.61 ~ 2.09)		
DA	190 ng/mL (< 20)	HbA1c	10.0% (4.9 ~ 6.0)		
PRA	0.6 ng/mL/h (0.2 ~ 2.3)	GA	29.2% (11 .0 ~ 16.0)		
PAC	< 10.0 ng/dL (4.0 ~ 82.1)	CRP	1.38 mg/dL (0.00 ~ 0.14)		
DHEA-S	119 μg/dL (5 ~ 253)	BNP	261.9 pg/mL (< 18.4)		
F-testosterone	6.8 pg/mL (4.6 ~ 16.9)				

*Abbreviation*s: *AD* Adrenaline, *NAD* Noradrenaline, *DA*, dopamine, *PRA* Plasma renin activity, *PAC* Plasma aldosterone concentration, *DHEA-S* Dehydroepiandrosterone sulfate, *U-MT* Urinary metanephrinem *U-NMT* Urinary normetanephrine

**Fig. 2:**
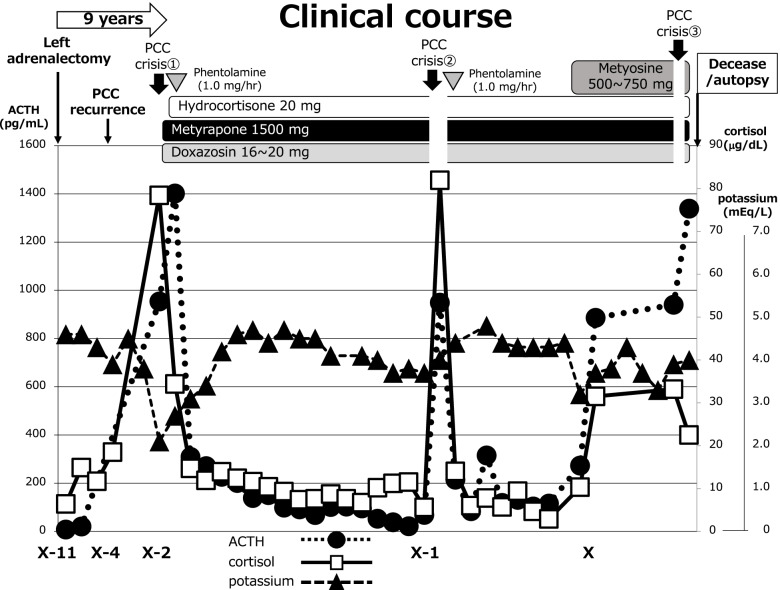
Clinical course of the patient. The clinical course of medical treatments, ACTH (closed circle), cortisol (open square), and potassium (closed triangle) in this case are indicated

At diagnosis, urgent corrections of cortisol and blood pressure were necessary. Thus, metyrapone (1500 mg/day), phentolamine (1.0 mg/hr), and doxazocin (16 to 20 mg/day) were first administered (Fig. 2), and a fluid infusion containing appropriate levels of potassium was initiated. Soon after metyrapone administration, the levels of ACTH and cortisol were both decreased and hydrocortisone supplementation (20 mg/day, (10 mg, 5 mg, 5 mg)) was initiated to mimic the normal circadian variation of cortisol levels as suppression/replacement therapy (Fig. 2). Thus, the existence of positive feedback loops of ACTH/cortisol and catecholamine/cortisol were suspected. At first, insulin injections were necessary to control his glucose levels. Soon after initiation of metyrapone treatment, his glucose levels became stable and insulin injections were tapered, and insulin injections and potassium supplementation were no longer required along with the correction of hypercortisolemia. Additional metyrosine treatment (500–750 mg) at later stage successfully stabilized his physiological and biochemical conditions (Fig. 2).

**Fig. 3:**
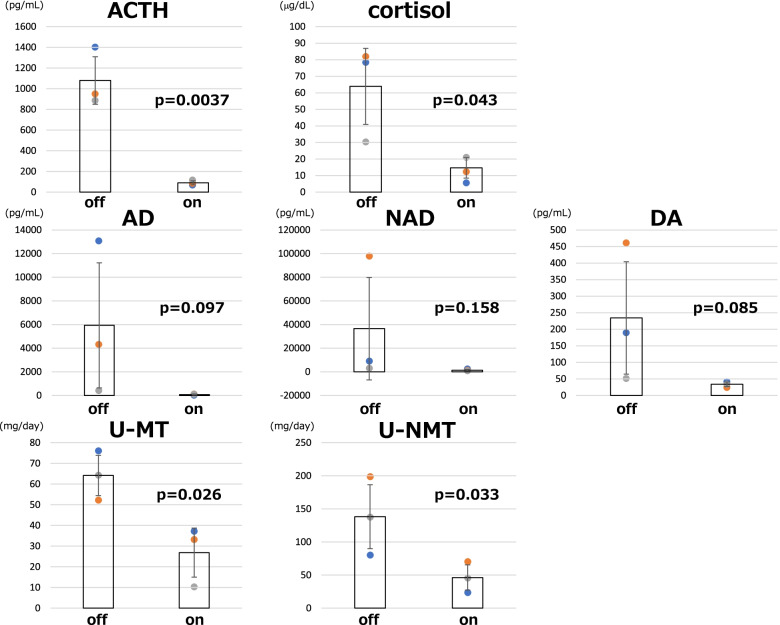
The off-to-on effects of metyrapone treatment on ACTH, cortisol, AD, NAD, DA, U-MT, and U-NMT. The numerical values of biomarkers were extracted upon three independent PCC crises including the first development of Cushing symptoms. The values of metyrapone-off and metyrapone-on were compared using paired *t*-test. AD, adrenaline; NAD, noradrenaline; DA, dopamine; U-MT, urinary metanephrine; U-NMT, urinary normetanephrine

During the clinical course, the patient was repeatedly hospitalized at our institution and was transferred to other hospitals several times. He experienced PCC crises at least three times with the complaints of nausea, appetite loss, and hypertension. When oral administration of metyrapone was not possible because of PCC crises, counter upregulation of ACTH and catecholamines were observed (Fig. 2). After these crises were controlled, the resumption of metyrapone treatment effectively reduced both ACTH and catecholamines. Changes in ACTH (1079.1 ± 229.8 ➔ 89.5 ± 19.7 pg/mL, 91.7% reduction; *p* = 0.0037), cortisol (63.9 ± 23.0 ➔ 14.6 ± 6.2 μg/dL, 77.1% reduction; *p* = 0.043), AD (5931.0 ± 5294.3 ➔ 78.0 ± 42.2 pg/mL, 98.7% reduction; *p* = 0.097), NAD (36,558.7 ± 43,399.0 ➔ 1406.3 ± 659.2 pg/mL, 96.2% reduction; *p* = 0.158), DA (234.3 ± 169.9 ➔ 34.0 ± 7.0 pg/mL, 85.5% reduction; *p* = 0.085), U-MT (64.1 ± 9.7 ➔ 26.8 ± 11.9 mg/day, 58.2% reduction; *p* = 0.026), and U-NMT (138.1 ± 48.2 ➔ 46.2 ± 19.1 mg/day, 66.6% reduction; *p* = 0.033) upon metyrapone treatment were observed during PCC crises, indicating that there was significant suppression of hormones (ACTH, cortisol, U-MT, and U-NMT) and trends of suppression (AD, NAD, and DA) on metyrapone treatment (Fig. 3). Therefore, the existence of positive feedback loops in the ACTH/cortisol and catecholamines/cortisol axes was confirmed.

Because aggressive treatments such as surgical intervention or chemotherapy for ACTH-producing recurrent malignant metastatic PCC were not performed because of the growing tumor size (Fig. 1 E), adhesions into surrounding organs, possible rupture risk, and poor general performance status with pulmonary edema (Fig. 1 F), the patient gradually became sick and was re-admitted to our hospital this year. On day 3 of the admission, he suddenly became hypoglycemic (30–40 mg/dL) and pancytopenic (WBC 1300/μL (3300 ~ 8600); Hb 8.4 g/dL (13.7 ~ 16.8); Plt 4.6 × 10^3^/μL (158 ~ 348)), and developed atrial fibrillation. An urgent CT scan revealed right adrenal subcortical bleeding (Fig. 1, arrow G), but no evidence of rupture in left adrenal PCC (Fig. 1 G, double arrow. Maximum diameter was 140 mm). No emergency lifesaving measures were taken because of the large PCC tumor located close to the heart. He died on day 4 and a pathological autopsy was performed that day.

### Pathological autopsy

**Fig. 4:**
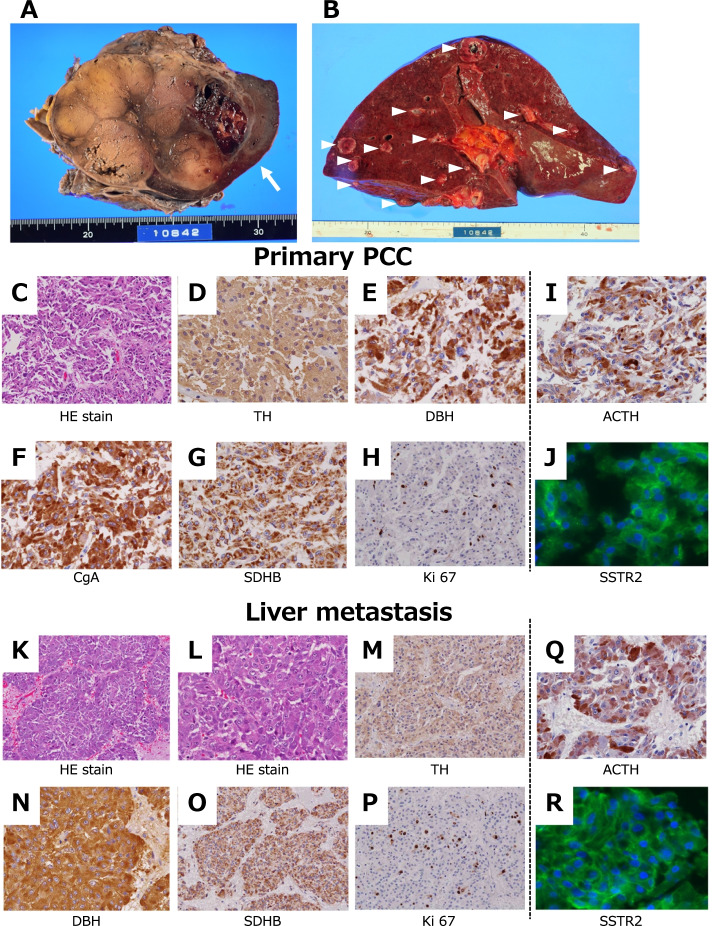
Representative autopsy samples and immunohistochemical investigations. **A**: Gross section of the primary PCC tumor. The arrow indicates deeply compressed spleen. **B**: Gross section of the liver. Arrowheads indicate metastatic lesions. C–R: HE staining and immunostaining with the corresponding antibodies indicated in the Fig. TH, tyrosine hydroxylase; DBH; dopamine-β-hydroxylase; CgA, chromogranin A; SDHB, succinate dehydrogenase B; SSTR, somatostatin receptor

A recurrent tumor of 120 mm at the largest diameter was located in the left retroperitoneal region that displaced the left kidney to the lower side and the spleen to the upper side, and invaded the pancreas and stomach (Fig. 4 A). Multiple liver metastases of up to 30 mm in size (Fig. 4 B) and paraaortic lymph node metastases were observed. The left adrenal gland was previously resected. The right adrenal gland was atrophic and could not be identified. Concentric left ventricular hypertrophy with marked heart weight gain (570 g) was observed and left ventricular myocardial thickening (15 mm) with systemic severe atherosclerosis suggested prolonged hypertension. Abscess formation was identified in the prostate and right peri-renal region causing peri-renal hemorrhage. Pulmonary congestion and edema were evident.

On the basis of the autopsy findings, the cause of the death was circulation and respiratory failure due to massive lung edema possibly caused by systemic infection and hypertensive cardiovascular disease. Several situations including a cancer-bearing condition, compromised infection and sepsis due to hypercortisolemia, subsequent disseminated intravascular coagulation (DIC), and hemodynamic instability due to catecholamine production from the tumor could have negatively contributed to the patient’s death.

### Histopathological examinations

Microscopic examinations with immunohistochemical analysis were performed for the tumor tissues of the left adrenal gland obtained by autopsy. The tumor consisting of tumor cells with hyperchromatic oval-shaped nuclei and basophilic granular cytoplasm was diffusely proliferated (Fig. 4 C). Post-mortem changes were remarkable in the intercellular voids. Immunostaining was positive for TH (Fig. 4 D), DBH (Fig. 4 E), CgA (Fig. 4 F), and SP (not shown), which corresponded to PCC. The expression of SDHB was preserved (Fig. 4 G). No S100-positive supporting cells were observed (not shown). The Ki 67 labeling index was 5.4% in the PCC (Fig. 4 H) and 8% in liver metastasis (Fig. 4 P). Similar histopathological and immunostaining patterns were observed in liver metastatic lesions (Fig. 4 K– P). Grading of the Adrenal Pheochromocytoma and Paraganglioma (GAPP) score [11] was 4 in both PCC and liver metastasis.

ACTH was strongly positive in the tumor cells of PCC (Fig. 4I) and in liver metastasis (Fig. 4 Q). Immunostaining of somatostatin receptors such as SSTR1, SSTR2, SSTR3, and SSTR5 was performed, and clear membrane localization of SSTR2 was evident in both PCC (Fig. 4 J) and metastatic lesions (Fig. 4 R). SSTR1, SSTR3, and SSTR5 were negative in both PCC and liver metastasis (not shown). Thus, the diagnosis of ACTH-producing PCC with liver metastasis was histologically and biochemically confirmed. Notably, most cells were TH-positive as well as ACTH-positive. These cells appeared to be mutually inclusive.

**Fig. 5:**
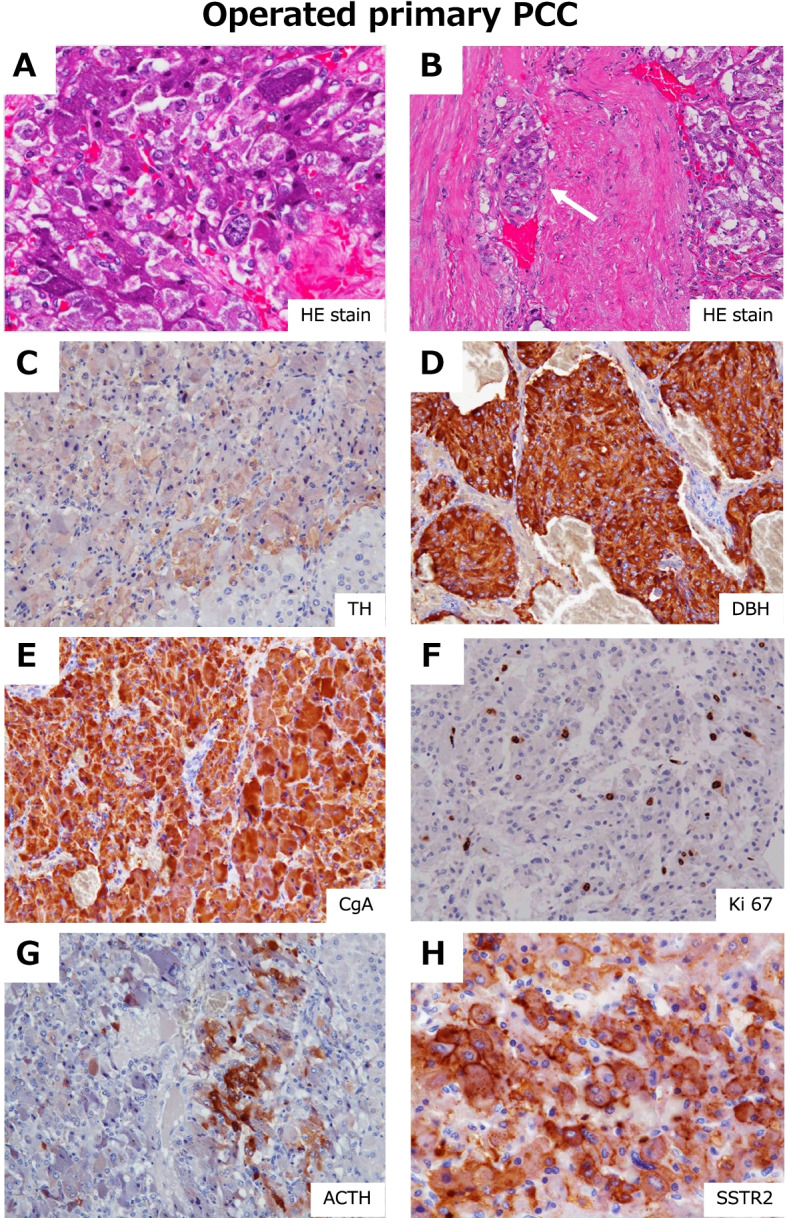
Immunohistochemical investigations of samples obtained during surgery 11 years previously. A–H: HE staining and immunostaining with the corresponding antibodies indicated in the figure

We also obtained the specimens from left adrenalectomy surgery performed 11 years previously. Similar microscopic examinations with immunohistochemical analysis were performed. Similar architectural characteristics in HE staining were observed as seen in Fig. 4 C (Fig. 5 A). Tumor infiltration was observed in small vessels (Fig. 5 B, arrow). Immunostaining was positive for TH (Fig. 5 C), DBH (Fig. 5 D), and CgA (Fig. 5 E), which corresponded to PCC, and the Ki 67 labeling index was 5.0% (Fig. 5 F). Focal staining of ACTH (Fig. 5 G) and intense expression of SSTR2 (Fig. 5 H) were observed. The GAPP score [11] was 4, suggesting that this tumor had preserved similar activity for 11 years. Although the biochemical activity of ACTH production was not evident at the time of initial surgery, a small amount of ACTH was suspected to be produced by the tumor since that time.

## Discussion and conclusions

PCC with simultaneous production of ectopic ACTH is a rare NET arising from the adrenal gland [2–4]. Our case indicated a large recurrent left adrenal PCC tumor with extremely high ACTH production at least 2 years after the recurrence of PCC.

The pathophysiological characteristics of this exceptional case can be explained by the confirmation of ACTH-producing PCC with glucocorticoid-regulated positive feedback loops [6, 7]. A parallel reduction not only in cortisol but also in ACTH and catecholamine levels upon metyrapone treatment (Figs. 2 and 3) were observed. The existence of positive feedback loops in the ACTH/cortisol and catecholamines/cortisol axes were confirmed by the off-to-on effects of metyrapone treatment (Fig. 3). It was also suggested that ACTH levels and the ACTH/cortisol ratio were extremely high in clinically diagnosed cases with positive feedback regulation [6–8]. In the present case, the highest ACTH level was 1402 pg/mL and the ACTH/cortisol ratio was 17.9 × 10^− 4^, indicating that this case may be analogous to the previously reported positive feedback cases [7]. When ACTH levels were higher than 200 pg/mL or an ACTH/cortisol ratio was more than 6 × 10^− 4^, an ectopic ACTH-producing tumor, regardless of whether positive feedback regulation exists, should bear in mind [7].

The transcriptional machinery of ACTH and catecholamines regulated by glucocorticoid has been proposed [7]. In general, glucocorticoids inhibit the production of proopiomelanocortin (POMC), an ACTH precursor, by binding to the glucocorticoid receptors, which translocate to the nucleus and interact with the negative glucocorticoid response element (GRE) of the *POMC* gene promoter. In ectopic ACTH-producing tumors, hypomethylation of the *POMC* promoter, particularly the E2F binding site, may be responsible for the paradoxical ACTH response to glucocorticoid [6]. It is also reported that glucocorticoid-dependent demethylation the *POMC* promoter has been shown in ACTH-producing thymic carcinoids [12]. Although we have not confirmed the hypomethylation of the *POMC* promoter or glucocorticoid dependent positive regulation of ACTH secretion in this particular case, the phenomenon of the off-to-on effects of metyrapone treatment were confirmed. It is speculated that the metyrapone treatment primarily reduced cortisol levels by 11β hydroxylase inhibition, and the reduced cortisol decreases glucocorticoid receptor binding on the putative hypomethylated E2F binding site in *POMC* promoter, thus finally reduces POMC/ACTH production in this case.

In fact, there was significant suppression of hormones (ACTH, cortisol, U-MT, and U-NMT) and trends of suppression (AD, NAD, and DA) upon metyrapone treatment (Fig. 3). The tentative diagnostic criteria of the presence of a positive feedback loop proposed by Inoue et al. [7] was the 80% reduction in ACTH upon metyrapone treatment. In our case, the reduction of ACTH on metyrapone was 91.7%, indicating that the response in this case could be compatible with the diagnosis of the presence of a positive feedback loop.

A positive feedback loop was also apparent in the catecholamine/cortisol axis. It is well known that glucocorticoids stimulate the synthesis of catecholamines by the induction of phenylethanolamine N-methyltransferase (PNMT) and TH [13, 14]. Moreover, catecholamines activate ACTH production through α-adrenergic receptors [15].

It seems that the glucocorticoid-centric positive feedback loops may explain the relatively rapid exacerbation of Cushing’s syndrome and PCC through two interacting vicious cycles, namely glucocorticoid-ACTH positive regulation and glucocorticoid-catecholamine positive regulation. Recent report suggests that there is a significant heterogeneity of the ectopic ACTH/CRH secreting PCC [16]. Single-cell transcriptome analysis indicates that there are many types of multiple hormone secreting cells in those ectopic ACTH/CRH secreting PCC, suggesting that regulations of hormone secretion in endocrine tumor may be more complicated [16]. Although this case presented hypercortisolemia with ACTH and catecholamine overproductions, the inter-relationship among those hormone regulations should be more precisely investigated e.g. in ex vivo analysis or others.

It should be noted that PCC crisis may be induced by dexamethasone suppression test [17], suggesting that combinations of diagnostic methods other than dexamethasone suppression test should be used when ACTH-producing PCC with positive feedback loops is suspected.

The levels of ACTH and catecholamines in our case were both critically high. The high hormone productive abilities may be explained by the dense ACTH, TH and DBH immunostaining and the large tumor size with positive feedback loops.

A review by Gabi et al. described the characteristics of 58 previously reported cases of ACTH-producing PCC from 1977 to 2017 [18]. After 2017, additional seven additional reports [5, 7, 19–23] described ACTH-producing PCC cases, and we have collected and analyzed the data of 65 cases in total.

The average age of patients with ACTH-producing PCC was 48.8 ± 13.5 years old (median, 49.5 years; range, 15–75 years). Only 13 cases were male (20%), and the remaining 52 cases (80%) were female with a significant difference (chi-squared test, *p* < 0.001). The average tumor diameter of ACTH-producing PCC was 4.42 ± 1.88 cm (median, 4.0 cm; range, 1.0–11.0 cm). According to these previous data, our case may be an extremely rare example presenting in an older (80 years) man, with the largest reported left adrenal tumor (14 cm on CT scan, 12 cm on autopsy). This large size of adrenal tumor may be due to the long-term follow-up period with no surgical intervention after recurrence. The ratio of the Ki 67 labeling index was 5.4% on autopsy, which is not that high, and was almost sustained for at least 11 years (5.0% at surgery; Fig. 5 F), suggesting that the tumor itself may not be severely aggressive but the long-term process may have enabled the tumor to enlarge.

Although there are some missing data in the literatures, the numerical values of ACTH, cortisol, and catecholamine were extracted from these 65 previously published reports describing ectopic ACTH production in PCC. Average ACTH was 358.9 ± 268.8 pg/mL (median, 289.1 pg/mL (range, 23.8–1157 pg/mL)), cortisol was 99.0 ± 78.0 μg/dL (median, 72.5 μg/dL (range, 24.6–339.4 μg/dL)), urine-free cortisol was 3898.4 ± 5110.8 μg/day (median, 2200 μg/day (range, 110.5–22,153.9 μg/day)), AD was 2079.4 ± 2160.2 pg/mL (median, 589.0 pg/mL (range, 48.2–4970 pg/mL)), NAD was 3663 ± 3777.4 pg/mL (median, 1342.0 pg/mL (range, 210.0–8901 pg/mL)), DA was 107.3 ± 68.0 pg/mL (median, 68.0 pg/mL (range, 51–203 pg/mL)), U-MT was 3.38 ± 2.27 mg/day (median, 2.70 mg/day (range 1.10–10.1 mg/day)), and U-NMT was 2.14 ± 1.48 pg/mL (median, 1.29 pg/mL (range, 0.67–4.8 pg/mL)). Our case presented the highest levels of ACTH (1402 pg/mL on admission), AD (13,071 pg/mL on admission), NAD (97,832 pg/mL on 2nd PCC crisis), DA (461 pg/mL on 2nd PCC crisis), U-MT (76.0 mg/day on admission), and U-NMT (197.8 mg/day 2nd PCC crisis) among those in 65 reported cases. These data support that the present case had extremely high hormone-producing abilities for ACTH and catecholamines, possibly due to the enormous size of the tumor and the positive feedback loops in the ACTH/cortisol and catecholamine/cortisol axes.

SSTRs are highly expressed in NET and have been targets for imaging and therapy. Somatostatin is a natural 14-amino-acid peptide hormone with regulatory effects in the endocrine system via binding to SSTR1–5, among which SSTR2 is a major target of the somatostatin receptor analogs (SRAs). Several types of SRAs have been developed for treatment and imaging, and ^177^Lu-DOTATATE (PRRT: peptide receptor radiotherapy) is now available for theranostic purposes [24]. Although SRA or PRRT was not approved as healthcare services provided by Japanese health insurance coverage, PRRT may have positive impacts in the treatment of inoperable PCC. PRRT may have potential advantages over conventional ^131^I-MIBG in terms of efficacy (tumor regression in 36%) and safety, such as hematopoietic toxicity [25]. The tumor control rate was reportedly 85% (partial response; 23% and stable disease; 67%) in a recent retrospective analysis [26].

SRS imaging was positive in the left adrenal PCC in our case, suggesting that the tumor may have co-produced ACTH as a NET, but was negative in liver metastatic lesions. Although SRS did not detect any metastatic lesions in the liver, immunohistochemical analysis indicated that SSTR2 was positive in both PCC and liver metastasis in this case. Although it could be recommended to analyze immunohistochemical staining to evaluate the individual grading of metastatic lesions, immunohistochemical examinations of SSTR2 had no advantage value compared to SRS uptake in predicting tumor response after PRRT [27].

Importantly, when PCC is suspected, the procedures of biopsy are not recommended.

In many ACTH-producing PCC cases, surgical removal of the tumor produces good prognostic results [18]. In our particular case, large tumor size and invasion into surrounding organs with poor physical status prevented us undertaking surgical intervention. In such cases, medication therapy should be selected to control hormone levels and/or hormone actions.

The inhibition of catecholamine synthesis by metyrosine (α methyl-tyrosine), which inhibits TH, is well known [28], and recently became clinically available in Japan. The administration of metyrosine at a later stage in our case rescued the severe symptoms derived from PCC crises. Anti-hypertensive medication, including α-blockers such as doxazocin, are widely used to inhibit catecholamine actions and are recommended at high doses.

Severe hypercortisolemia is characterized as a life-threatening emergent endocrine condition in patients with Cushing syndrome. Adrenal steroidogenesis inhibitors (etomidate (11β hydroxylase inhibitor), ketoconazole (17α and 11β hydroxylase inhibitor), or metyrapone (11β hydroxylase inhibitor), alone or in combination therapy, are commonly the first-line treatments for severe hypercortisolemia because of their rapid action, good efficacy, and safety profiles. Novel compounds such as osilodrostat (11β hydroxylase inhibitor), abiraterone (17α hydroxylase and 17, 20 lyase inhibitor), or efavirenz (21α hydroxylase inhibitor) are future potential candidates for the regulation of cortisol production [29]. A glucocorticoid receptor antagonist, mifepristone, has a rapid action as well, but its use has been limited because of difficulties in monitoring its efficacy and safety. Other slow-acting cortisol-lowering medications (mitotane, cabergoline, and pasireotide) might be candidates in the therapeutic options [30].

It is also proposed that rapid improvements in hypercortisolemia upon adrenal steroidogenesis inhibitor treatment in the presence of hypercatecholaminemia might be harmful because acute loss of the protective and life-saving effects of glucocorticoid may cause epinephrine shock [31]. In these cases, appropriate hydrocortisone supplementation, such as that undertaken in our case, may be necessary to avoid adrenal insufficiency.

In summary, we experienced the largest reported left adrenal recurrent metastatic PCC simultaneously producing ACTH in an older male. Pathophysiological analysis suggested that ACTH and catecholamine production is positively regulated by cortisol. Suppression of cortisol synthesis by metyrapone effectively reduced both ACTH and catecholamines, indicating that our case possessed a positive feedback loop in the ACTH/cortisol and catecholamine/cortisol axes, which may explain the extremely high levels of these hormones.

## Data Availability

Data are available on request to the authors.

## Abbreviations

PCC: Pheochromocytoma
TH: Tyrosine hydroxylase
NET: Neuroendocrine tumor
CgA: Chromogranin A
SP: Synaptophysin
DBH: Dopamine-b-hydroxylase
SDHB: Succinate dehydrogenase B
SSTR: Somatostatin receptor
AD: Adrenaline
NAD: Noradrenaline
DA: Dopamine
MT: Metanephrine
NMT: Normetanephrine
MIBG: Metaiodobenzylguanidine
SRS: Somatostatin receptor scintigraphy
DIC: Disseminated intravascular coagulation
GAPP: Grading of the Adrenal Pheochromocytoma and Paraganglioma
PMNT: Phenylethanolamine N-methyltransferase
SRA: Somatostatin receptor analog
PRRT: Peptide receptor radionuclide therapy
